# Demobilisation or Resurrection in Ireland

**Published:** 1919-01-11

**Authors:** 


### THE FATE OF VOLUNTARY WAR WORKERS.

### Demobilisation or Resurrection in Ireland.

NOTHING throughout the war has been more remarkable than the silent devotion of British women to the men-at-arms in the fighting line. Their number has been legion, their work of priceless value, and yet, withal, the public know so little of its volume, or of its intrinsic value, that it is scarcely an exaggeration to say that they know nothing. In the Metropolis, in the big cities of the kingdom, and in forgotten hamlets, women of high rank and humble have toiled with tireless energy making things for the wounded. Bandages and dressings, dressings and bandages—these have been supplied by the million. In addition, papier mâché splints, made for nothing and almost out of nothing, with infinite patience and scientific skill, slippers, treasure bags, and a score of other hospital accessories, have appeared as though by magic.

### A SANCTIFIED SERVICE.

Where the material came from, how and where the various articles were manufactured, who inspired and organised the toilers—all this is as much a mystery to the man in the street as though the information had been suppressed by the Censor. It is literally true to say that this colossal task has been a thankless one. Neither the municipality nor the State has so far expressed a word of gratitude to the workers. Nevertheless, these women have reaped a rich reward in knowing in their own souls that they toiled not in vain. They entered on their duties knowing that theirs was a sanctified service. They played to no gallery, desiring only to share in common with the soldier and the sailor, and to the best of their ability, the burden of the conflict.

### SUMMARY DISMISSAL?

The question arises, what is to become of these women? Are the doors of the War Hospital Supply Depôt to be closed with a bang and the workers summarily dismissed? It is just possible that the question may never be put, and that the blinds will be pulled down unnoticed. In the dignity of silence the work has been carried on, and now that the war is over, its value and importance are scarcely likely to be advertised. For lack of public appreciation, and for lack of leadership, this vast volume of potential energy is likely to be allowed to drift. Indeed, if immediate and vigorous action is not taken, this must happen. In many districts the process of waste is now in progress.

### WHAT IRELAND HAS ACCOMPLISHED.

In Ireland a very practical proposition has been made by Mrs. Dorothy Hignett, O.B.E., one of the most energetic and practical war workers in the kingdom. Mrs. Hignett is an Englishwoman who has made Ireland her home, and since the tragic day that war was declared she has devoted her entire time and infinite energy to the cause. She began with munition canteens, and went to Woolwich to learn the details and routine of procedure under Lady Lawrence. This knowledge acquired, she returned to Dublin, and, under the presidency of Lady Waterford, organised bands of voluntary canteen workers for all the National Shell Factories in the country. There is not a detail of this work, including buying, carving, and distributing, of which Mrs. Hignett is not master. Then began the work of organising War Hospital Supplies, initiated in Ireland by Lady Waterford. This was a huge task of propaganda. The women of Ireland had to be aroused, organised, and instructed. Suitable premises had to be found in towns, villages, and in out-of-the-way places miles from a railway station, and every worker had to be taught, not alone how to prepare the equipment, but the fundamental necessity for scrupulous cleanliness to the point of sterilisation, not alone with the material, but with the workroom and its furniture. Some idea of the work done in the provinces of Leinster, Munster, and Connaught, under the presidency of Lady Waterford, and her deputy, Mrs. Hignett, may be gathered from the fact that upwards of £60,000 worth of war hospital supplies were dispatched to Red Cross Hospitals at the various battle fronts and at home. Considering that the population of the territory referred to is sixty per cent. lower than that of Lancashire, this output is astonishing.

### IRISH HOSPITALS AND MRS. HIGNETT.

This digression is unavoidable, and is to show that in a sparse and scattered population every available ounce of energy has been utilised, and the question arises, is it to be allowed to drift? It is now proposed by Mrs. Hignett that this splendid organisation shall not be demobilised, but shall continue its labours permanently for the benefit of the civil hospitals of this country. This extremely practical suggestion originated with Miss Overend, one of the most active and earnest workers at 40, Merrion Square, the Headquarters and Central Depôt of Ireland, who correctly interpreted and expressed the common desire to continue such useful and patriotic labour. The idea is a brilliant one, and needless to say the hospitals have received the proposal with delight. Many of these institutions are very poor, and even with the best off the linen department is a constant source of anxiety. Apart, however, from the direct and immediate benefit which a central supply establishment must confer, there are other considerations which may not have occurred to the authors. An immense accession of public interest in local hospitals must accrue through the medium of these bands of voluntary workers labouring for their welfare. Moreover, under a possible and prospective Ministry of Health, the co-operation of such workers will be invaluable.

### IRELAND'S BOOK OF PROGRESS.

From whatever standpoint the subject is viewed it seems obvious that immediate and vigorous effort should be made to prevent demobilisation. The great armies of healers that have been organised for war are bound to be of equal value in peace. There is infinite scope for their services in carrying into effect future schemes of reconstruction and reform, and if they are allowed to scatter it is extremely doubtful that it will be possible to gather them together again. "IERNE."

